# Allocation of the “Already” Limited Medical Resources Amid the COVID-19 Pandemic, an Iterative Ethical Encounter Including Suggested Solutions From a Real Life Encounter

**DOI:** 10.3389/fmed.2020.616277

**Published:** 2021-01-14

**Authors:** Yazan Nedal Alhalaseh, Hatem A. Elshabrawy, Madiha Erashdi, Mohammed Shahait, Abdulrahman Mohammad Abu-Humdan, Maysa Al-Hussaini

**Affiliations:** ^1^Department of Internal Medicine, King Hussein Cancer Center, Amman, Jordan; ^2^Department of Molecular and Cellular Biology, College of Osteopathic Medicine, Sam Houston State University, Conroe, TX, United States; ^3^Department of Pathology and Laboratory Medicine, King Hussein Cancer Center, Amman, Jordan; ^4^Department of Surgery, King Hussein Cancer Center, Amman, Jordan; ^5^Independent Researcher, Amman, Jordan; ^6^Human Research Protection Program Office, King Hussein Cancer Center, Amman, Jordan

**Keywords:** COVID-19, resources, low-income countries, challenges, solutions

## Abstract

The shortage of healthcare providers is well-documented in low-income countries (LIC) prior to COVID-19, due to various causes including the migration to developed countries, scarcity of supplies, poor healthcare infrastructure, limited ICU facilities, and lack of access to guidelines and protocols. One of the important hitches in LIC is the insufficient testing capacity that precluded accurate assessment of disease burden and subsequently resource allocations. Trying to adhere to the principles of bioethics including respect to others, beneficence, and justice should be applied on the ground in the particular setting of the LIC. Solutions should be tailored to the tangible needs and possibility of implementation in real life in the face of the “already” limited resources by making use of simple, yet plausible, measures. Implementing guidelines and frameworks that were set to work in the better-resourced nations is a call for futility. The adoption of novel solutions to overcome the unique challenges in the LIC is exigent. These include the use of automated screening algorithms and virtual video clinics. Moreover, integrating electronic intensive care unit (e-ICU) software may allow for remote monitoring of multiple patients simultaneously. Telemedicine could help in getting consultations worldwide. It can also enhance healthcare workers' knowledge and introduce new skills through teleconferences, e-workshops, and free webinars. Healthcare workers can be remotely trained to enhance their skills. Agencies, such as the WHO, should develop comprehensive programs to tackle different health issues in LIC in collaboration with major institutions and experts around the world.

## Introduction

Coronaviruses (CoVs) are a family of positive single-stranded RNA (_+_ssRNA) viruses that belong to family *Coronaviridae* (1). Three out of seven human coronaviruses (HCoVs) cause severe respiratory diseases of high fatality rates (2, 3). The first is Severe Acute Respiratory Syndrome-CoV (SARS-CoV) that emerged in 2002 followed by Middle East Respiratory Syndrome-CoV (MERS-CoV) in 2012 (2). In December 2019, SARS-CoV-2 was identified as the third HCoV that causes acute respiratory distress syndrome (ARD) with viral pneumonia (3). This disease was later named COVID-19 (4). The first cases of SARS-CoV-2 infections originated in the city of Wuhan, China and soon the disease spread to 177 countries causing a global outbreak (4). Millions of COVID-19 cases and more than a million deaths have been reported worldwide. The World Health Organization (WHO) had declared a public health emergency and characterized COVID-19 as a global pandemic on March 11, 2020 (5). SARS-CoV-2 positive cases with varying disease severity have flooded hospitals and healthcare facilities. The number of cases and deaths varied per country depending on the protective measures and resources to deal with such a highly transmissible and infectious virus.

The main concern in COVID-19 pandemic is that the disease burden may exceed healthcare resources that are available for treating patients (6). Even in developed countries, there was a concern that healthcare systems would be overwhelmed if COVID-19 cases increase dramatically (6). For example, in USA, there were not enough N95 masks which necessitated the reuse of such single use masks (7, 8). In Italy, ventilators and ICU beds were made available only for critically ill patients during the peak of the disease (9, 10). South Korea faced a shortage in hospital beds which lead to many deaths (10, 11).

Healthcare systems in developing countries face major problems during this time and are unlikely to offer the care needed. The scarcity in healthcare resources, training, and low number of healthcare workers are the most important reasons (10). Developing countries lack the testing capacities and the technologies to trace the infected individuals. Moreover, the cost of the COVID-19 screening test in developing countries mostly exceeds the total sum that healthcare systems spend per individual. N95 masks are in short supply in many developing countries. According to the United Nations, there are only an average of 113 hospital beds per 100,000 in developing countries which is 80% lower than the number in developed countries (12). Moreover, developing countries have a scarcity of ICU beds (0.1–2.5 per 100,000) when compared to developed countries (5–30 beds per 100,000) (13).

The scarcity of healthcare resources, particularly in developing countries, may create ethical dilemmas. This may include the need to provide care and treatment for more severely ill patients while delaying treatment for others who are in a better condition (14). The need to take such decisions may cause some healthcare workers to experience moral injury or mental health problems (15). It becomes very challenging when such decisions have to be made at the expense of ethical values.

In this article, we will discuss the deleterious impacts of the COVID-19 pandemic on healthcare workers and availability of resources, particularly in countries with limited resources, and will provide possible solutions to cope with the current emergency.

## Methodology

We did a literature search to identify challenges facing healthcare workers in countries with limited resources during COVID-19 pandemic. We discuss the number of physician per capita, the number of hospital beds, ICU and ventilators in limited settings, the allocation of limited budget to the healthcare system and its impact on other services and more prevalent health conditions. Finally, we provide insight into possible solutions that may help alleviate the stress and demand on healthcare system, resources, and personnel.

### Challenges Faced by the Healthcare Systems in LIC

#### Healthcare Workers Shortage and Burnout

A disparity between different countries' response to COVID 19 pandemic is evident. According to the World Bank data (Table 1), high and high-intermediate income countries have a higher number of physicians and nurses per capita as compared to low and low-intermediate income countries. Shortage of physicians in countries with insufficient resources could be attributed to slow economic growth that leads to limited healthcare annual budget and exodus of physicians to work in higher income countries. A study conducted by Astor et al. investigated the factors that contributed to physician migration from developing to developed countries. The desire for increased income, greater access to enhanced technology, need for safer, more stable, and better future for the family were the main listed causes (24). Another study discussed the insufficient numbers of surgeons, obstetricians and anesthesiologists in low- and intermediate income countries which was in part due to lack of training and educational opportunities for surgeons and other healthcare workers (25–27). The shortage of healthcare workers and scarcity of resources in low- and intermediate- income countries have led to increased work hours and burnout of healthcare personnel in these countries and more severe economic deterioration.

**Table 1:** The medical resources available in low income countries, low-intermediate, high-intermediate countries in comparison with high-income countries.

	**Low**	**Low-intermediate**	**High-intermediate**	**High**
Income (July 2019/ $)^+^	<1,026	1,026–3,995	3,996–12,375	>12,375
Number of countries (2020) ^+^	29	50	56	82
Physicians/10,000 population (2017)^+^	3	8	20	31
Nurses and midwifery/10,000 population (2018)^+^	9	18	35	109
Hospital beds/ 1,000 population ^+^ Examples: ^*^	0.8 (2006) 0.87 (2017) (Bangladesh)	1 (2011) 1.38 (2017) (Mexico)	3.5 (2012 ) 8.05 (2017) (Russia)	4.2 (2013) 8.00 (2017) (Germany)
ICU beds/100,000 population ^*^	0.72 (Bangladesh) (16)	1.2 (Mexico) (17)	8.3 (Russia) (18)	38.7 (Germany) (19)
Ventilators^*^	No data (Bangladesh)	2,050 (Mexico) (20)	40,000 (Russia) (21)	25,000 (Germany) (22)
Total expenditure on health Per capita (PPP int. $) (2017)^+^	44.8	80.5	459.9	5284.1
Gross national income per capita g (PPP int. $)/(2019)^+^	791.8	2189.4	9074	45307.3
Cellular phone subscriber (per 100 population) (2018)^+^	60.8	94.3	117.3	127.6

+ *The data in these rows were referenced from world bank data (23)*.

* *The data in these rows were taken from different references (16–22)*.

In the 2013–2016 Ebola outbreak, studies showed that stress and anxiety, due to tremendous pressure on healthcare workers, could lead to faster spread of the disease and the probability of healthcare workers quitting their job (28). This could result in a healthcare system collapse.

The healthcare systems worldwide are dealing with pandemic-related challenges and stressors that could eventually lead to healthcare workers' burnout (14). These include the fear of spreading infection to family members, and others, due to the close interaction with COVID-19 patients, increased workload, and requirement to provide care and treatment for all critically ill patients in the setting of inadequate PPE and other resources (14). This may require treatment of more severely ill patients while delaying treatment for others who are in a better condition; decisions which may cause some healthcare workers to experience moral injury or mental health problems. This may potentially progress to mental health problems such as depression, post-traumatic stress disorder, and even suicide (15). Furthermore, reduced social support, lack of self-care and family time, and lack of information about the COVID-19 transmission and disease prognosis are all factors that add up to the stressors that healthcare workers have to face (29). During the last SARS-CoV outbreak in Guangdong, China, there were reports of stress, anxiety, depression, and general psychological stress among health professionals (30). In addition, 21% of SARS cases were reported among healthcare providers (30).

#### Exhausted Healthcare Systems and Limited Testing Capacity

LIC have long been challenged by the limited healthcare resources including the availability of ICU and mechanical ventilators, even before the pandemic. The high cost of mechanical ventilators, the need for proper training and education, and the unavailability of ventilator protocols are factors that contributed to the challenges facing healthcare systems in developing countries (31). Unfortunately, the militarization of healthcare in some of the LIC, particularly in areas of conflicts and wars, adds to the limitation. The ongoing wars in some of LICs have put these countries in a more compromised situation (32). Patients suffering from chronic diseases have also suffered from decreased follow up to their conditions with others not even getting diagnosed due to the overwhelming of the health sector. For example, Skeete et al. found that patients with hypertension have suffered worse outcomes (higher mortality and morbidity) during the ongoing pandemic (33).

**Figure 1:**
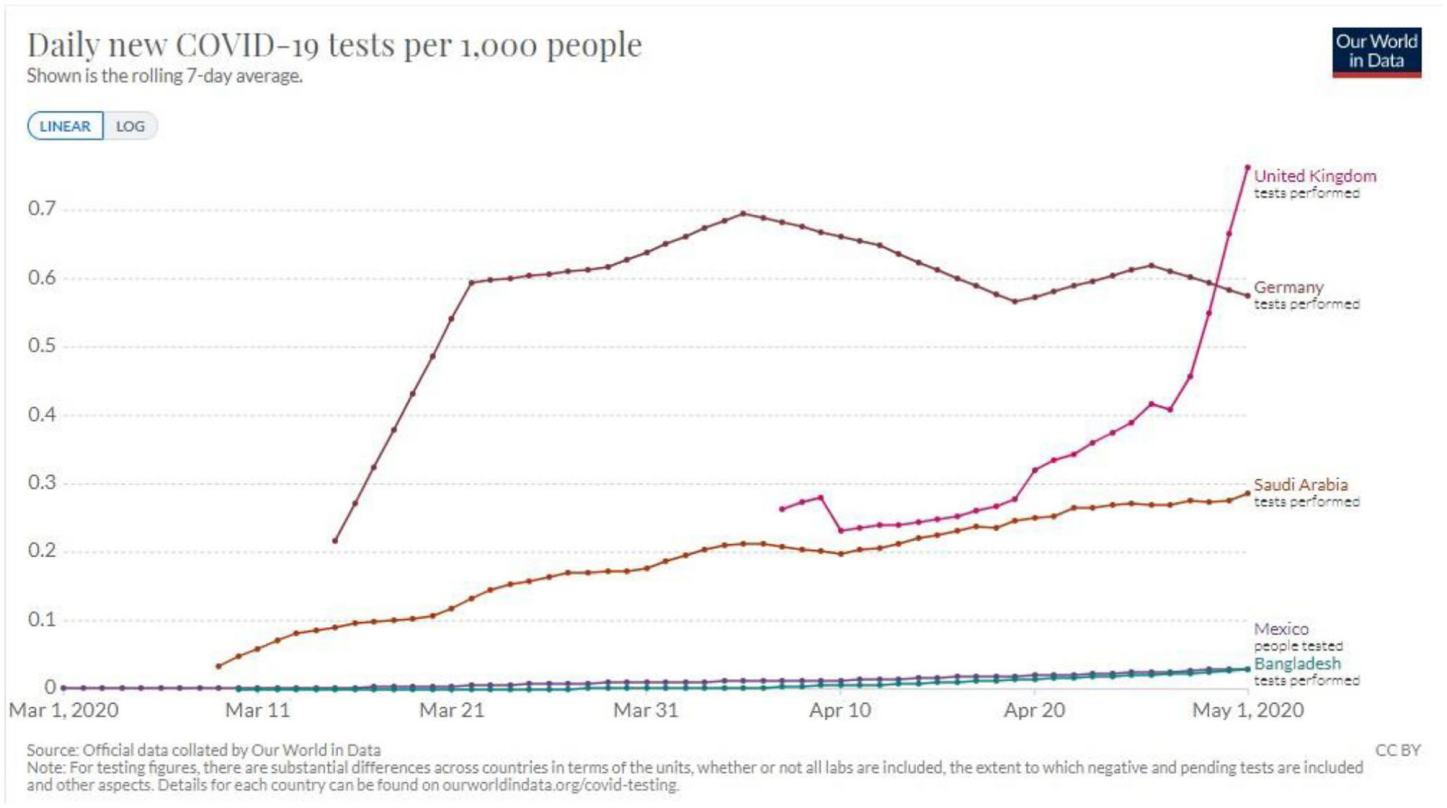
Testing capacity variations among countries of different incomes during the beginning of the pandemic (34).

**Figure 2:**
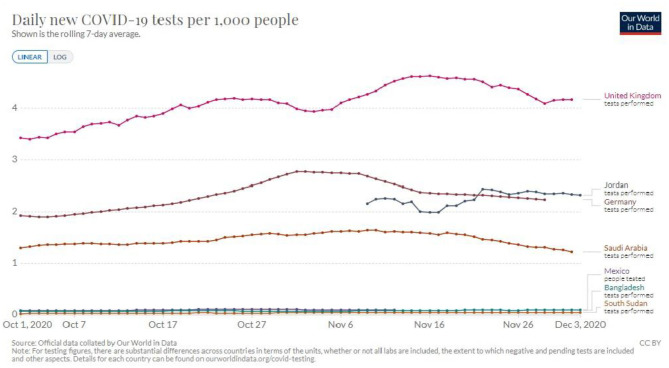
Testing capacity variations among countries of different incomes during the period of October–December, with a focus on Jordan's testing capacity in comparison to higher income countries (34).

In addition, the shortage of testing capacity in LIC has left most people untested, thus precluding the accurate estimation of disease burden (Figure 1). Consequently, low testing capacity has led to inadequate planning to maximize the use of the available healthcare resources (35–37). Without enough data, countries would not be able to estimate the disease burden leading to poor allocation of resources toward combating the pandemic. In Jordan, for example, the government opted for a complete and strict lockdown at the beginning of the pandemic. Then gradually these measures were loosened causing a delay in the first major wave till mid to late October. During this time the government was able to ramp up its testing capabilities and have testing be wildly available and immediately act when the number of COVID-19 cases started increasing in October (Figure 2).

#### The Scarcity of Personal Protective Equipment (PPE)

Disposable filtering face piece respirators (FFRs) including N95 respirator and surgical masks are designed for one-time use. LICs have always struggled with a deficient supply of Personal Protective Equipment (PPE). In view of the increased demand and shortage of supply, even with donations, stocks remained insufficient (35, 38, 39). Accordingly healthcare workers are wearing these masks for an extended period of time or they consider reusing them (40).

#### Humanitarian Aid Challenges

The withdrawal of humanitarian organizations‘ personnel, and travel constraints that interfere with international aids all add to the challenges that LICs have to face (35, 41, 42). During a crisis, the need for humanitarian aid spikes in LIC. This spike is normally dealt with by providing quick response, from logistical hubs, to supply the countries in need with the necessary provisions. However, during the lock downs and travel restrictions, aimed to suppress the spread of the virus, imposed by the countries that contain these hubs has left the countries dependent on aid in a dire need (43).

### Suggested Solutions Tailored to the Actual Needs and Applicability to the LIC

#### Increasing Healthcare Systems' Work Force

Asking retired health care professionals to join the workforce to fight COVID-19, is an option adopted by some health authorities. Waiver of medical licensing fees and expediting the renewal of licenses for healthcare professionals, while providing extensions for the licenses that are expiring soon, could assist in promoting the accruing of this group of physicians. This might help in reducing the load on the practicing doctors. Retired healthcare workers are experienced, knowledgeable, and emotionally stable. However, asking retired healthcare professionals who are in their 60s and beyond to provide direct patient care could pose risk of infections for such individuals. We believe that it is better for this particular group of healthcare professionals to provide telemedicine or support services. Additinally, physicians who are specialized in other clinical areas may be trained to work in intensive care units. Medical assistants and nurses may be asked to help in patients' treatments under physicians' supervision.

Asking medical students of different institutions to help according to the level of seniority might also alleviate some of the workload. Medical students are usually young individuals who might be better suited to support the medical teams. We believe that these stratigies may increase the number of physicians at emergency care eventually leading to a reduced workload on individual physicions.

#### Social Support of the Healthcare Frontline Workers

In LIC, the extended families play a pivotal role in supporting working familiy members. However, practicing healthcare workers might be living away from their families. It is important that healthcare system leadership take measures to ensure that the healthcare workers are fully supported and cared for. Physicians who are placed on quarantine or are working over extended hours should be offered care for their children. Alternatively they could be asked to do office work for those who are in clinics and hospitals, and provide remote care and consultation for patients. Additional institutional policies that may help in alleviating healthcare workers stress and burnout may include paid time off and sick days in addition to coverage of expenses for employees with COVID-19-related illnesses.

#### Telemedicine

The application of telemedicine can be a sustainable solution to many of our challenges during the COVID 19 pandemic. According to the European commission, it can be defined as the provision of healthcare by using electronic information and communication technology to securely transmit information in text, sound, images and other forms to prevent, diagnose, or treat patients (44). Given the novelty of the COVID 19 virus, telemedicine can offer the community with a trusted medical opinion and avoid the chaos created by unreliable information on social media (45). It can help with the spread of experiences and medical knowledge form different parts of the world to reach remote areas and underserved communities by conducting frequent conferences and meetings between healthcare professionals and improving the response to this healthcare emergency (45). With the large increase in smartphone and internet users in developing countries, programs, and phone applications that allow for remote patient-doctor interaction are widely available (46). This can be very quickly utilized by training healthcare professionals to conduct online consults, as these applications don't require much training for use (46, 47).

Between October 5 and October 26, an online virtual workshop, organized by the WHO, took place. In this workshop, medical health professionals from the Jordanian public health sector were given lectures on various topics pertinent to COVID-19 screening and patient management protocols. This online workshop facilitated the sharing of medical knowledge and protocols while at the same time setting the trend of online lecturing and data sharing. These methods were then implemented in various hospitals in the public sector, thus facilitating a better quality of health care while limiting the need for large gatherings (48).

Another major benefit is the provision of direct interaction between patients and healthcare professionals. This helps maintain follow-up on chronic diseases from a distance, which might be interrupted due to fear from acquiring the infection (45). Moreover, it can maximize the granted benefit from limited resources and physicians by enhancing the efficacy of critical care services and making it possible to expand ICUs with the same number of physicians by allowing off site intensivist to monitor patients in multiple locations simultaneously (49). Consultations from worldwide experts through many freely available applications on the readily available personal mobile phones can enhance healthcare workers knowledge and introduce new skills by teleconferences, e-workshops, and free webinars. Healthcare workers can be remotely trained to enhance their skills.

In King Hussein Cancer Center in Jordan various methods of limiting unnecessary exposure were implemented. Patients were contacted, by phone, by nurse coordinators from the center before scheduled appointments. In these phone calls, patients were asked if they suffered from any symptoms that would suggest a COVID-19 infection. They were also asked if they had been in contact with a confirmed COVID-19 case. Screening tests and vital signs were taken for patient with a high degree of suspicion before being admitted for scheduled procedures. Upgrades were also made to an existing call center to facilitate remote clinics (50).

#### Mobile Applications in Tracking Possible COVID-19 Patients

LIC have limited resources to allocate to testing a large number of people, so it is important to develop ways to maximize the efficiency of the testing process (Figures 1, 2). Mobile applications that use location to determine the proximity of a person to an affected individual can help in contact tracing, ultimately maximizing the efficiency of testing. These applications, if used by a large enough number of people, along with social distancing measures, could be sufficient in slowing the progress of the pandemic to a manageable rate. Similar measures have been deployed in Mainland China and South Korea with great success (51).

#### The Use of 3D Printing as a Possible Solution for Limited Equipment Due to Lack of Resources

The shortage in the medical supply chain has triggered us to search for bright solutions that can reshape our future response to persistent challenges. The technology of 3D printing is evolving and has a wide variety of applications. It has the ability to produce anything anywhere and can adapt complex manufacturing instruction in a short time and at a lower cost. During the current pandemic, it was used to manufacture PPE including facemasks, face shields, and goggles. Some universities used 3D printing to create diagnostic tools such as microscopes. In china, 3D printers were used to create quarantine booths (52). Moreover, 3D printing firms are volunteering their expertise and skills to respond to the current crises. Many 3D printing companies such as Stratasys, Carbon, and Shape ways are working rapidly to produce ventilator components, face masks, and medical test equipment. To globally materialize 3D printing service, they have shared free files for a 3D printed add-on hands-free door handles (52). These add-ons allows users to open most modern doors using their elbows to avoid touching door handles (hotspot for microbes) that are subjected to a lot of physical contact, especially in public places such as offices and hospitals (52).

## Conclusion

This pandemic has presented a unique challenge to developed and developing countries alike. LIC countries have faced a harder toll due to preexisting challenges in their healthcare systems. Despite this there are many ways to utilize existing infrastructure to help combat the pandemic by utilizing retired healthcare workers, using telemedicine, or taking advantage of cheaper technologies (such as 3D printing) to decrease the burden of the pandemic. Raising public awareness remains of pivotal importance to decrease the pressure of the pandemic on the health care system. Public health measures known to limit viral spread are highly encouraged, these include hand hygiene, cough etiquette and social distancing; as they will reduce the need for limited supplies (10). We also believe that the WHO, as a global health oversight board, should develop comprehensive programs to tackle the different issue facing healthcare systems in LIC. Emergency health funding should be offered to enable health authorities in LIC to purchase appropriate consumables for healthcare workers.

## Author Contributions

MA-H: the conception of the idea. YA, HE, ME, MS, and AA-H: literature review. YA, HE, and AA-H: writing the first draft. ME, MS, AA-H, and MA-H: critical review. All authors: final approval. All authors contributed to the article and approved the submitted version.

## Conflict of Interest

The authors declare that the research was conducted in the absence of any commercial or financial relationships that could be construed as a potential conflict of interest.
